# Risk of immune-related diarrhea with PD-1/PD-L1 inhibitors in different cancer types and treatment regimens

**DOI:** 10.7150/jca.32724

**Published:** 2020-01-01

**Authors:** Lei Zhao, Jing Yu, Jing Wang, Huihui Li, Juanjuan Che, Bangwei Cao

**Affiliations:** Cancer center, Beijing Friendship Hospital, Capital Medical University, Beijing 100050, China

**Keywords:** Cancer, Diarrhea, Randomized controlled trials, PD-1/PD-L1 inhibitors, CTLA-4 inhibitor, Chemotherapy

## Abstract

**Objective:** To compare the incidence and severity of diarrhea among different tumor types and treatment regimens, and also compared with CTLA-4 inhibitors in randomized controlled trials.

**Methods:** MEDLINE, PMC database and EMBASE were retrieved until December 2018. Studies were eligible if they were randomized controlled trials and included participants undergoing PD-1/PD-L1 inhibitors for cancer, measured a treatment side effect of diarrhea, and reported quantitative data. The risks of diarrhea in PD-1/PD-L1 inhibitors were compared among different treatment regimens.

**Results:** Totally 21 studies involving 11554 patients were included for meta-analysis. For all-grade diarrhea, the risk after the PD-1/PD-L1 inhibitors plus CTLA-4 inhibitor combination was 1.90 times significantly higher than that of monotherapy, and the risk was 0.69 and 0.60 times significantly lower than that of monotherapy compared with chemotherapy and ipilimumab. The incidence of diarrhea was not significantly different between PD-1/PD-L1 inhibitor monotherapy versus placebo or between low-doses versus high-doses. For high-grade (grade ≥ 3) diarrhea, the risk after the PD-1/PD-L1 inhibitors plus CTLA-4 inhibitor combination was 3.29 times significantly higher than that of monotherapy, the risk in PD-1/PD-L1 inhibitors monotherapy was 0.50 and 0.38 times significantly lower than chemotherapy and ipilimumab respectively. No significant difference was found in the incidence of diarrhea between PD-1/PD-L1 inhibitor monotherapy versus placebo or between low-doses versus high-doses whether in all-grade or high-grade group.

**Conclusions:** PD-1/PD-L1 inhibitors have a lower risk of developing diarrhea than chemotherapy and CTLA-4 inhibitor. There is no direct relationship between the dose of PD-1/PD-L1 inhibitors and the risk of developing diarrhea.

## Introduction

Increasing evidence proves the significant efficacy of immune checkpoint inhibitors (ICIs) in treatment of advanced cancers 1-4. ICIs targeting the programmed cell death protein 1/programmed death ligand 1 (PD-1/PD-L1) pathway significantly improve the progression-free survival and overall survival compared with standard chemotherapy, so PD-1/PDL1 antibodies are currently approved for treatment of various malignancies 5-11. Since the anti-PD-1 antibody pembrolizumab was approved in September 2014 for treatment of advanced melanoma, the clinical development of PD-1/PD-L1 inhibitors as anticancer drugs has been widely expanded. Currently, the Food and Drug Administration has approved PD-1/PD-L1 inhibitors for treatment of 9 types of cancers. For instance, pembrolizumab can be used to treat melanoma 2, 12-14, non-small cell lung cancer (NSCLC) 7, 15-19, head and neck squamous cell carcinoma (HNSCC) 20, Hodgkin's lymphoma 21, urothelial cancer 22, 23 and gastric cancer 24. Anti-PD-1 antibody nivolumab is recommended for treating melanoma 11, 25, renal cell carcinoma (RCC)^26^, Hodgkin's lymphoma 27, 28, urine epidermal cancer 29, MSI-H colon cancer 30 and hepatocellular carcinoma 31. Anti-PD-L1 antibody atezolizumab is suggested for treatment of urothelial cancer 22, 32 and NSCLC 6, 33, and anti-PD-L1 antibodies avelumab and durvalumab can be used to treat urothelial cancer^34^, ^35^. Compared with cytotoxic chemotherapy, ICIs have unique toxicity based on their action mechanism, which is considered to be immune-related adverse event (IRAE) 36-39. Inhibiting the PD-1/PD-L1 pathway may lead to autoimmune toxicity, some of which may be severe or even life- threatening 36, 40.

Diarrhea is a common side effect of cancer treatment that, in severe cases, can lead to death or to patients having to stop lifesaving treatment because often there are no effective therapies to control the diarrhea. Diarrhea in cancer patients can quickly lead to life-threatening consequences such as dehydration, electrolyte imbalance, shock, etc. Compared to chemotherapy-related diarrhea the immunological preparation of PD-1/PD-L1 is prone to cause autoimmune digestive diseases such as ulcerative colitis, and may also cause side effects of diarrhea.

Given the clinical efficacy evidence for a wide spectrum of tumor types, the PD-1 ICI therapy is expected to be increasingly used by oncologists as a monotherapy or in combination with other drugs. Therefore, physicians in cancer immunotherapy must be familiar with the pathogenesis of diarrhea in different tumors and different treatment regimens, and provide useful information to optimize the management of this toxicity. At present, there is no complete description about the clinical experience of anti-PD-1/PD-L1-associated diarrhea patients, or about the management and outcome of this toxicity. Therefore, we conducted a meta-analysis of PD-1 inhibitors in cancer patients and compared the incidence and severity of diarrhea among different tumor types, different treatment regimens.

## 1. Methods

### 1.1. Literature selection and data extraction

Two researchers (Lei Zhao and Huihui Li) independently reviewed the databases Medline, PMC database and EMBASE to select potential relevant articles. Any discrepancy between them was resolved by consensus. The following medical subject heading terms were used: PD-1, PDL1, CD274, programmed death receptor 1, programmed death receptor ligand, immune checkpoint inhibitor, nivolumab, BMS936558, pembrolizumab, MK-3475, MPDL3280A, atezolizumab, avelumab, MSB0010718C, durvalumab, and diarrhea. The databases were searched from the inception until December, 2018.

The inclusion criteria were: (a) phase I, II and III trials in cancer patients; (b) random assignment of participants to single PD-1/PD-L1 inhibitor treatment or other control therapy (e.g. ipilimumab, placebo); (c) reporting diarrhea events or event rate and sample size for any all-grade or high-grade (≥3) adverse events;(d) random controlled trial.

The following information was extracted by two independent reviewers (Lei Zhao and Huihui Li) from the included studies: first author, publication year, study name, clinical trial registration number, total number of patients, mean age, trial phase, treatment plan, tumor type, primary inclusion criteria, and numbers of patients with all grades and high-grade treatment-related diarrhea. The treatment regimens were classified as PD-1/PD-L1 inhibitor monotherapy, PD-1/PD-L1 inhibitor plus CTLA-4 inhibitor ipilimumab, chemotherapy, placebo, and ipilimumab. According to the different doses, monotherapy was divided into low-dose group and high-dose group.

### 1.2. Quality assessment

The two reviewers (Lei Zhao, Huihui Li) used the Jadad scoring method^41^ to evaluate the quality of each included study from randomized (0 or 1), double-blind (0, 1 or 2), recorded loss of follow-up and/or exit (0 or 1) and assign hidden (0 or 1). A score ≥ 3 indicates high quality.

### 1.3. Statistical analysis

Meta-analysis for statistical analysis was performed using Stata12.1 (Stata Corp, College Station, TX, USA). Heterogeneity was analyzed by Q test, and I^2^<25%, 25%-75%, and >75% indicate mild, moderate and significant heterogeneity, respectively. In case of insignificant heterogeneity between studies indicated as P>0.05, a fixed effect model was used; otherwise a random effect model was used. The incidence of diarrhea was evaluated by relative risk (RR) and 95% confidence interval (CI), and the analysis results were represented by forest maps. Two-tailed p < 0.05 was considered significant. This meta-analysis has been registered on the PROSPERO website (Registration Number: CRD42018111834).

## 2. Results

**Figure 1:**
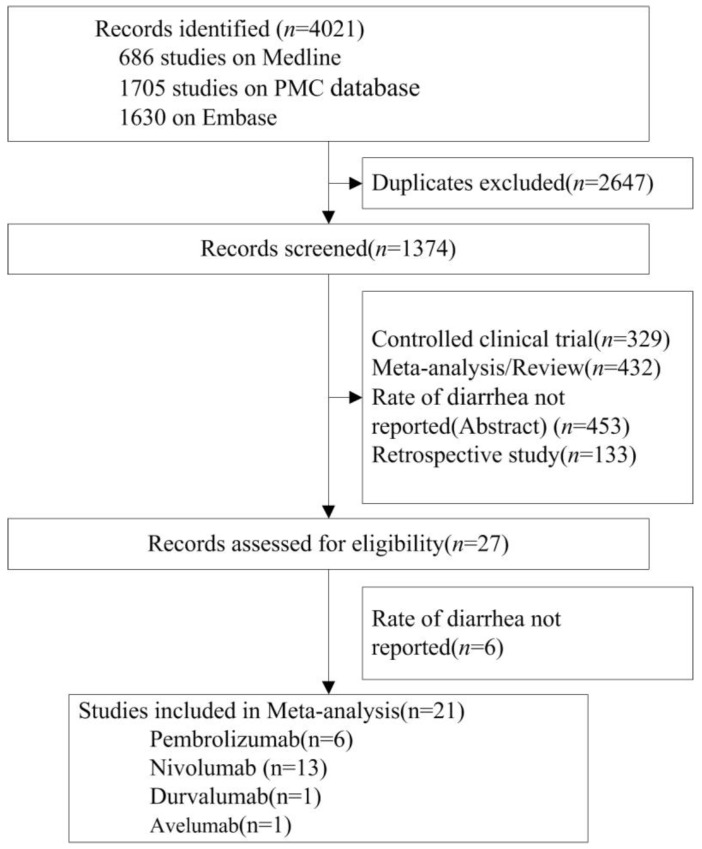
Flow Diagram of Study Inclusion.

The database search initially returned 4021 studies. After screening and eligibility assessment, a total of 21 randomized controlled trials (RCTs, n=11554 patients) were identified for meta- analysis. ICIs tested in these studies included nivolumab (n=13 studies), pembrolizumab (n=6), avelumab (n=1) and durvalumab (n=1). Tumor types tested included NSCLC (n=7 studies), melanoma (n=10), HNSCC (n=1), Renal Cell Carcinoma (RCC) (n=1), gastric cancer (n=1) and small cell lung cancer (SCLC) (n=1). According to the clinical staging, 15, 5 and 1 of the 21 RCTs were at phase 3, 2 and 1, respectively (Table 1, Figure 1).

### 2.1. Risks of diarrhea among different treatment regimens

#### All-grade diarrhea

**Figure 2:**
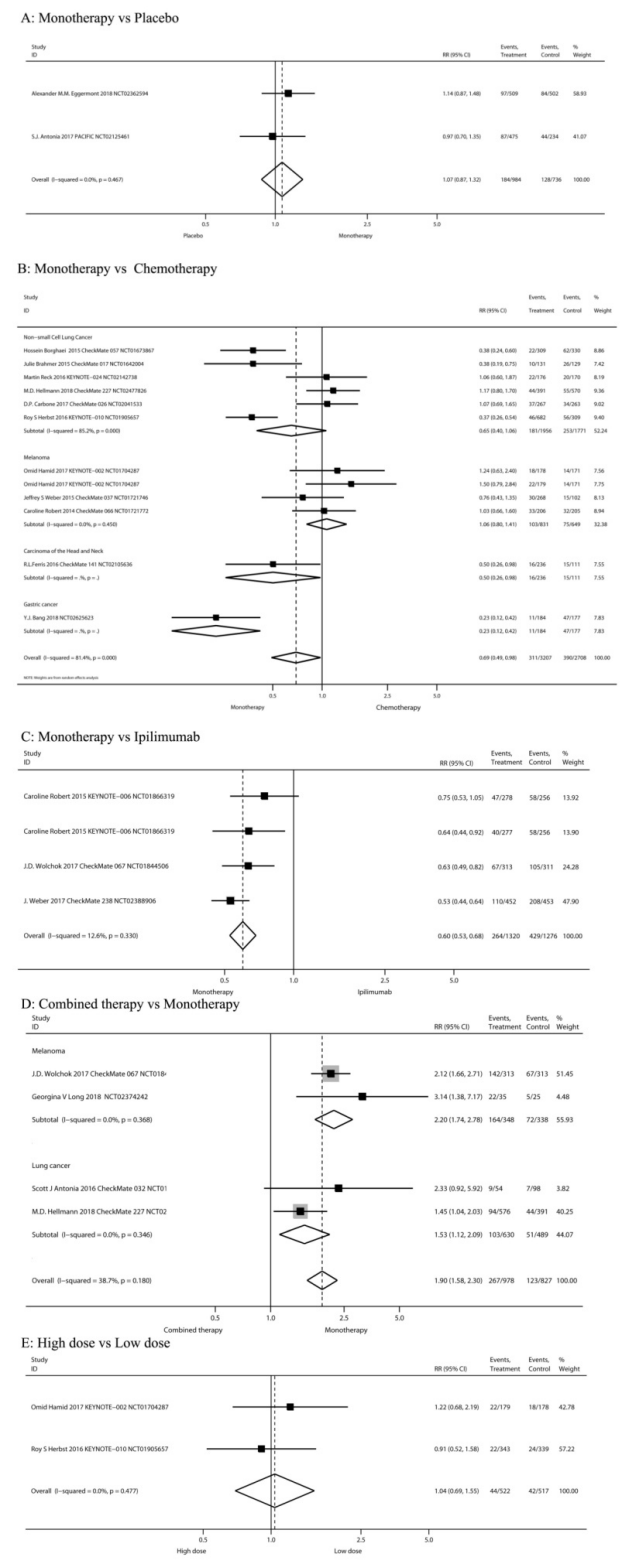
The risks of all-grade diarrhea in PD-1/PD-L1 inhibitors were compared among different treatment regimens.

The risks of all-grade diarrhea in PD-1/PD-L1 inhibitors were compared among different treatment regimens: PD-1/PD-L1 inhibitor monotherapy versus PD-1/PD-L1 inhibitor plus ipilimumab, versus chemotherapy, versus placebo, versus ipilimumab, and high-dose versus low-dose in the 20 studies (Figure 2).

##### PD-1/PD-L1 inhibitor monotherapy versus placebo

Two RCTs 42, 43 compared PD-1/PD-L1 inhibitor monotherapy and placebo (n=1,720 patients). Classification based on tumor type included melanoma (n = 1 study) and NSCLC (n = 1). Classification according to the use of ICIs included pembrolizumab (n = 1) and durvalumab (n = 1). The pooled RR of all-grade diarrhea incidence was not significant after PD-1/PD-L1 inhibitor monotherapy (RR 1.07, 95%CI: 0.87-1.32, P=0.516) (Figure 2A).

##### PD-1/PD-L1 inhibitor monotherapy versus chemotherapy

Eleven RCTs 5, 7, 11, 12, 18, 44-49 compared PD-1/PD-L1 inhibitor monotherapy and conventional chemotherapy (n=5,915 patients). Classification according to tumor type included melanoma (n = 3 study), HNSCC (n=1), Gastric cancer (n=1) and NSCLC (n = 6). Classification according to the use of ICIs included nivolumab (n = 7 study), pembrolizumab (n = 3) and Avelumab (n=1). The risk of all-grade diarrhea after PD-1/PD-L1 inhibitor monotherapy was significantly decreased (RR 0.69, 95%CI: 0.49-0.98, P=0.037; Figure 2B). The risk of all-grade diarrhea after PD-1/PD-L1 inhibitor monotherapy in Carcinoma of the Head and Neck and Gastric cancer patients were significantly decreased (RR 0.50, 95%CI: 0.26-0.98, P=0.043; RR 0.23, 95%CI: 0.12-0.42, P=0.000 Figure 2B).

##### PD-1/PD-L1 inhibitor monotherapy versus ipilimumab

Three RCTs 13, 50, 51 compared PD-1/PD-L1 inhibitor monotherapy and ipilimumab (n=2,596 patients). The tumor type was malignant melanoma. Classification according to the use of ICIs was nivolumab (n=2) and pembrolizumab (n=1). The study group of Caroline Robert 2015^13^ was divided into two subgroups according to drug intervals and thus can be analyzed as two studies. The pooled RR of all-grade diarrhea incidence after the PD-1/PD-L1 inhibitor monotherapy (nivolumab or pembrolizumab) was significantly decreased (RR 0.60, 95%CI: 0.53-0.68, P=0.000; Figure 2C).

##### Nivolumab plus ipilimumab compared to nivolumab monotherapy

Four RCTs 42, 48, 50, 52 compared nivolumab plus ipilimumab and nivolumab monotherapy (n= 1,805 patients). Classification of tumor type was SCLC (n=1 study), NSCLC (n=1) and melanoma (n=2). Our meta-analysis reveals that nivolumab plus ipilimumab significantly increased the risk of all-grade diarrhea compared to nivolumab monotherapy (RR 1.90, 95%CI: 1.58-2.30, P=0.000; Figure 2D).

##### High-dose group versus low-dose group

Three RCTs 7, 26, 47 compared the low-dose and high-dose PD-1/PD-L1 inhibitor monotherapy (n= 1039 patients). Pembrolizumab 2 mg/kg is defined as low dose; 10 mg/kg is high-dose. Nivolumab ≤2 mg/kg is defined as low dose, 10 mg/kg is high-dose. Classification according to tumor type was melanoma (n = 1 study), RCC (n = 1) and NSCLC (n = 1). Results showed no significant risk in the high-dose group (RR 1.15, 95%CI: 0.80-1.67, P=0.446; Figure 2E).

#### High-grade diarrhea

**Figure 3:**
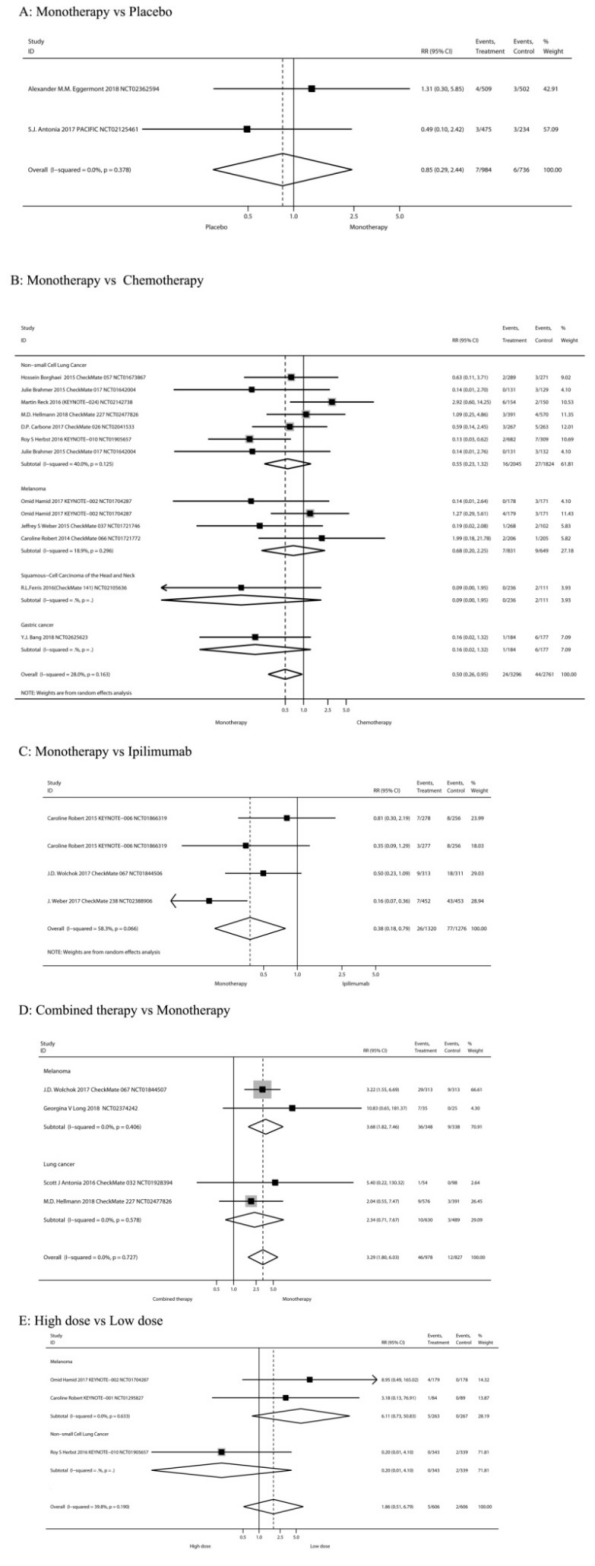
The risk of high-grade (≥ 3) diarrhea according to different treatment regimens.

Figure 3 showed the risk of high-grade (≥ 3) diarrhea according to different treatment regimens: PD-1/PD-L1 inhibitor monotherapy versus PD-1/PD-L1 inhibitor plus ipilimumab, versus chemotherapy, versus placebo, versus ipilimumab, and low-dose versus high-dose.

##### PD-1/PD-L1 inhibitor monotherapy versus placebo

Inclusion of the study and number of patient's high-grade diarrhea were consistent with previous all-grade diarrhea. As shown in Figure 3A, when compared with placebo, there was not a significant increase in the risk of high-grade diarrhea incidence for PD-1/PD-L1 inhibitor monotherapy (RR 0.85, 95%CI: 0.29-2.44, P=0.756).

##### PD-1/PD-L1 inhibitor monotherapy versus chemotherapy

Inclusion of the study and number of patient's high-grade diarrhea were consistent with previous all-grade diarrhea. Results showed a significant decreased in the risk of high-grade diarrhea after monotherapy (RR 0.50, 95%CI: 0.26-0.95, P = 0.035; Figure 3B). Of the 7 RCTs on non-small cell lung cancer, 5 were treated with nivolumab and 2 with pembrolizumab. The use of nivolumab or pembrolizumab seems to reduce the risk of diarrhea compared to chemotherapy, but the results are not significant (RR 0.58, 95%CI: 0.25-1.31, P = 0.190; RR 0.61, 95%CI: 0.03-13.01, P = 0.754, respectively).

##### PD-1/PD-L1 inhibitor monotherapy versus ipilimumab

Inclusion of the study and number of patients of high-grade diarrhea were consistent with previous grades of diarrhea. Results showed significantly decreased in the risk of high-grade diarrhea after PD-1/PD-L1 inhibitor monotherapy (RR 0.38, 95%CI: 0.18-0.79, P=0.009; Figure 3C). Our meta-analysis reveals that PD-1 antibodies (pembrolizumab or nivolumab) reduce the risk of severe diarrhea compared to ipilimumab.

##### Nivolumab plus ipilimumab compared to nivolumab monotherapy

Inclusion of the study and number of patients of high-grade diarrhea were consistent with previous grades of diarrhea. The tumor type was melanoma in all cases. Results showed no significant increase in the risk of high-grade diarrhea after nivolumab plus ipilimumab treatment (RR 3.29, 95%CI: 1.80-6.03, P=0.000; Figure 3E).

##### High-dose group versus low-dose group

Three RCTs 7, 12, 47 compared the High-dose and Low-dose treatments (n=1,212 patients). Classification of tumor type was melanoma (n=2 studies) and NSCLC (n=1). Results showed no significant increase in the risk of high-grade diarrhea after high-dose treatment (RR 1.86, 95%CI: 0.51-6.79, P=0.345; Figure 3F).

#### Study quality and publication bias

Fifteen trials were open label, whereas five trials were double blind controlled. The Jadad score ranged from 3 to 4. For RR of all-grade between PD-1/PD-L1 inhibitor monotherapy and chemotherapy or high-grade diarrhea between the monotherapy and ipilimumab, the Egger test suggested some evidence of publication bias. No evidence of bias was found in other comparisons of Egger tests (all P>0.05), or in all Begg tests (all P>0.05).

## 3. Discussion

Although an increasing number of clinical studies have confirmed the overall survival benefit of PD-1/PD-L1 inhibitors treatment, PD-1/PD-L1 inhibitors therapy increases the toxicity of the drug remains controversial, especially diarrhea. Our meta-analysis is the first large-scale analysis of different immunologic treatment regimens compared with chemotherapy or ipilimumab for the toxic side effects of diarrhea. In our research, the risk of all-grade diarrhea after the PD-1/PD-L1 inhibitors plus CTLA-4 inhibitor combination was 1.90 times higher than that of PD-1/PD-L1 inhibitors monotherapy (P<0.05), and the risk was 0.72 and 0.60 times higher than that of chemotherapy and ipilimumab compared with monotherapy (P<0.05). Chemotherapy is the most prone to diarrhea, followed by PD-1/PD-L1 inhibitors plus CTLA-4 inhibitor combination, and finally PD-1/PD-L1 inhibitors monotherapy. When compared with placebo, we did not observe a significant increase in the risk of all-grade or severe diarrhea incidence, and our meta-analysis also reveals that high-dose PD-1/PD-L1 inhibitor monotherapy did not increase the risk of all-grade or severe diarrhea when compared with low-dose(all P>0.05), which suggested that PD-1/PD-L1 inhibitor monotherapy is relatively safe. The risk of grade ≥ 3 diarrheas for PD-1/PD-L1 inhibitors alone significantly decreased than chemotherapy or ipilimumab, while the PD-1/PD-L1 inhibitors plus CTLA-4 inhibitor combination significantly increase than monotherapy.

The basic principle of binding PD-1 / PD-L1 inhibitors and CTLA-4 inhibitors is that they have different mechanisms of action. Anti-CTLA-4 mainly acts on the lymph node area, restores the induction and proliferation of activated T cells, and resists PD-1 acts mainly on the periphery of the tumor site, preventing the tumor-infiltrating tumor-infiltrating PD-L1-expressing tumor and plasma-like dendritic cells from neutralizing cytotoxic T cells 53. Our result found that patients taking CTLA-4 inhibitors ipilimumab had a significantly higher risk of developing diarrhea than those using PD-1 / PD-L1 inhibitors. This is likely to be related to the different mechanisms of action of the two drugs. Although the combination of PD-1/PD-L1 inhibitors plus CTLA-4 inhibitor has achieved good efficacy 25, 50, 52, the corresponding toxic side effects of combination therapy, especially diarrhea, are significantly higher than those of PD-1/PD-L1 inhibitors alone. Combination therapy with both CTLA-4 and PD-1 blockers raised the risk of GI toxicities to about 45% which is much higher than monotherapy 54. The risk of diarrhea was significantly different compared to the use of PD-1/PD-L1 inhibitors alone and chemotherapy in different tumor types. In patients with NSCLC patients, the risk of diarrhea using PD-1 / PD-L1 inhibitors monotherapy is significantly lower than that of patients receiving chemotherapy. However, this result did not find in melanoma patients. Our results show that patients taking pembrolizumab or nivolumab have a slightly different risk of all-grade diarrhea compared with chemotherapy 11, 12, 47. Furthermore, our study demonstrates no significant difference in the incidence of diarrhea between low-dose versus high-dose PD-1/PD-L1 inhibitors, which are consistent with another study 12. This provides reliable evidence for further exploration of adjusting drug doses in future clinical trial design and clinical practice.

As far as we know, this is the systematic review including the largest number of RCTs for analysis of immune-related diarrhea. The present study has some limitations. Firstly, these relevant studies have applied PD-1/PD-L1 inhibitors to different treatment lines, and there may be inconsistencies in the underlying characteristics of the patients. Secondly, since the present study is based on a secondary analysis of the final results of each report, we were unable to obtain patient-level disease characteristics and variables, or to determine the specific risk factors associated with the development of immune-related diarrhea. Thirdly, our results were influenced by the limitations of individual clinical trial design. Some of the clinical trials included in the meta- analysis were open label, which may lead to subjective bias. Finally, clinical RCTs included in the meta-analysis had strict inclusion and exclusion criteria. The patients selected in the study were with good PS, but in clinical practice, a large number of patients suffered impaired organ dysfunction and/or functional status and may have a higher incidence of actual toxicity. In the future, large-sample RCTs are needed to compare the incidence and severity of PD-1/PD-L1 inhibitor associated diarrhea among more tumor types and among more combination regimens.

Overall, PD-1/PD-L1 inhibitors have a lower risk of developing diarrhea than chemotherapy and CTLA-4 inhibitor. There is no direct relationship between the dose of PD-1/PD-L1 inhibitors and the risk of developing diarrhea. This study provides reliable evidence for further exploring the combination of PD-1/PD-L1 inhibitors with other drugs in clinical trial design and clinical practice.

## Figures and Tables

**Table 1:** Characteristics of Relevant Studies

Analysis Method	Source	Format	Data Set	Tumor Type	Main Inclusion Criterion	Treatment	Sample Size	Age, Median(range),y	Jadad score
Meta-analysis Meta-analysis	R.L.Ferris 2016 CheckMate 141 NCT02105636	Full text	Randomized, open-label, phase 3 trial	Head and neck	Recurrent squamous-cell carcinoma of the head and neck	1. Nivolumab 3 mg/kg Q2W 2. Standard therapy	240 121	59(29-83) 61(28-78)	3
	Julie Brahmer 2015 CheckMate 017 NCT01642004	Full text	Randomized, open-label, international, phase 3 study	NSCLC	Stage IIIB or IV squamous-cell NSCLC	1. Nivolumab 3 mg/kg Q2W 2. Docetaxel 75 mg/m2 Q3W	135 137	62 (39-85) 64 (42-84)	3
	Caroline Robert 2015 KEYNOTE-006 NCT01866319	Full text	Randomized, controlled, phase 3 study	Melanoma	Unresectable stage III or IV melanoma	1. Pembrolizumab 10 mg/kg Q2W 2. Pembrolizumab 10 mg/kg Q3W 3. Ipilimumab 3 mg/kg Q3W	279 277 278	61 (18-89) 63 (22-89) 62 (18-88)	3
	Omid Hamid 2017 KEYNOTE-002 NCT01704287	Full text	Randomised, open-label, phase 2 study	Melanoma	Unresectable stage III or stage IV melanoma not amenable to local therapy	1. Pembrolizumab 2 mg/kg Q3W 2. Pembrolizumab 10 mg/kg Q3W 3. Chemotherapy(carboplatin , carboplatin plus paclitaxel, dacarbazine, paclitaxel alone or oral temozolomide)	180 181 179	62 (15-87) 60 (27-89) 63 (27-87)	3
	J.D. Wolchok 2017 CheckMate 067 NCT01844505	Full text	Double-blind, Randomised, phase 3 trial	Melanoma	Stage III (unresectable) or stage IV melanoma	1.Nivolumab 1 mg/kg Q3W plus ipilimumab 3 mg /kg Q3W for four doses,followed by nivolumab 3mg/kg Q2W 2.Nivolumab 3 mg/kg Q2W 3.Ipilimumab 3 mg/kg Q3W for four doses	314 316 315	61 (18‒88) 60 (25‒90) 62 (18‒89)	4
	Michael A. Postow 2015NCT01927419	Full text	Randomized 2:1 in a double-blinded phase 2 trial	Melanoma	Unresectable, previously-untreated, stage III or IV melanoma with measurable disease	1. Nivolumab 1mg/kg Q3W plus ipilimumab 3mg/kg first 4 doses,then nivolumab 3mg/kg Q2W 2.Ipilimumab 3mg/kg first 4 doses	95 47	64 (27- 87) 67 (31- 80)	4
	Georgina V Long 2018NCT02374242	Full text	Multicentre, open-label randomised, phase 2 trial	Melanoma	Melanoma brain metastases	1. Nivolumab 1 mg/kg + ipilimumab 3 mg/kg Q3W for four doses; then nivolumab 3 mg/kg Q2W 2. Nivolumab 3 mg/kg Q2W 3. Nivolumab 3 mg/kg Q2W( brain metastases)	35 25 16	59 (53-68) 63 (52-74) 51 (48-56)	3
	Caroline Robert, 2014KEYNOTE-001 NCT01295827	Full text	Open-label, international, multicentre phase 1 trial	Melanoma	Progressive, measurable, unresectable melanoma	1.Pembrolizumab 2 mg/kg Q3W 2.Pembrolizumab 10 mg/kg Q3W	89 84	57 (18-88) 60.7 (27-86)	3
	Alexander M.M. Eggermont 2018 NCT02362594	Full text	Randomized, double-blind phase 3 trial	Melanoma	Resected stage III melanoma	1. Pembrolizumab 200 mg Q3W for 18 doses 2. Placebo	514 505	54 (19-88) 54 (19-83)	4
	S.J.Antonia 2017 PACIFIC NCT02125461	Full text	Global, randomized, phase 3 trial	NSCLC	Stage III, locally advanced, unresectableNSCLC	1. Durvalumab10 mg/kg Q2W for up to 12 months. 2. Placebo	476 237	64(31-84) 64(23-90)	3
	Martin Reck 2016 KEYNOTE-024 NCT02142738	Full text	Open-label, randomised , phase 3 trial	NSCLC	Untreated advanced NSCLC with PD-L1 expression on at least 50% of tumor cells kinase gene	1. Pembrolizumab 200 mg Q3W for 35 cycles 2.Investigator's choice of platinum based chemotherapy for 4 to 6 cycles	154 151	64.5(33-90) 66(38-85)	3
	D.P. Carbone 2017 CheckMate 026 NCT02041533	Full text	Open-label randomized phase 3 trial	NSCLC	Untreated stage IV or recurrent NSCLC and a PD-L1 tumor-expression level of 1% or more	1. Nivolumab 3 mg/kg Q2W 2. Platinum-based chemotherapy Q3W for up to six cycles	271 270	63(32-89) 65(29-87)	3
	Roy S Herbst 2016 KEYNOTE-010 NCT01905657	Full text	Randomised, open-label, phase 2/3 study	NSCLC	Previously treated non-small-cell lung cancer with PD-L1 expression on at least 1% of tumour cells	1. Pembrolizumab 2 mg/kg Q3W 2. Pembrolizumab 10 mg/kg Q3W 3. Docetaxel 75 mg/m² Q3W	344 346 343	63 (56-69) 63 (56-69) 62 (56-69)	3
	Scott J Antonia 2016 CheckMate 032 NCT01928394	Full text	Randomised,Cohort of this phase 1/2 multicentre, multi-arm, open-label trial	Recurrent small-cell lung cancer	Limited-stage orextensive-stage SCLC, and had disease progression after at least one previous platinum-containing regimen	1. Nivolumab 3 mg/kg Q2W 2. Nivolumab 1 mg/kg plus ipilimumab 3 mg/kg Q3W for four cycles, nivolumab3 mg/kg Q2W 3. Nivolumab 3 mg/kg plus ipilimumab 1 mg/kg Q3W for four cycles, nivolumab3 mg/kg Q2W	98 61 54	63 (57-68) 66 (58-71) 61 (56-65)	3
	M.D. Hellmann 2018 CheckMate 227 NCT02477826	Full text	Randomised,Multipart, open-label phase 3 trial	stage IV or recurrent NSCLC	stage IV or recurrent NSCLC that was not previously treated with chemotherapy	1. Nivolumab 3 mg/kg Q2W plus Ipilimumab 1 mg/kg Q6W 2. Nivolumab 240 mg Q2W 3. Chemotherapy	576 391 570	-	3
	Hossein Borghaei 2015 CheckMate 057 NCT01673867	Full text	Randomised, phase 3 study	Non-squamous NSCLC	stage IIIB/IV or recurrent non-squamous NSCLC	1. Nivolumab 3 mg/kg Q2W 2. Docetaxel 75 mg/m2 Q3W	292 290	61(37-84) 64(21-85)	3
	Jeffrey S Weber 2015 CheckMate 037 NCT01721746	Full text	Randomised, controlled, open-label, phase 3 trial	melanoma	unresectable or metastatic melanoma, and progressed after ipilimumab, or ipilimumab and a BRAF inhibitor if they were *BRAF*V^600^ mutation-positive	1. Nivolumab 3 mg/kg Q2W 2. ICC(dacarbazine 1000 mg/m^2^ Q3W; carboplatin area under the curve 6+ paclitaxel 175 mg/m^2^ Q3W)	272 133	59 (23-88) 62 (29-85)	3
	Caroline Robert 2014 CheckMate 066 NCT01721772	Full text	Randomised, double-blind, phase 3 trial	Melanoma	unresectable, previously untreated stage III or IV melanoma without a BRAF mutation	1. Nivolumab(3 mg/kg Q2W)+ placebo Q3W 2. Dacarbazine(1000 mg/ m2 Q3W)+ placebo Q2W	210 208	64 (18-86) 66 (26-87)	4
	J. Weber 2017 CheckMate 238 NCT02388906	Full text	Randomized, double-blind, phase 3 trial	Melanoma	complete resection of stage IIIB,IIIC, or IV melanoma	1. Nivolumab 3 mg/kg Q2W 2. Ipilimumab 10mg/kg Q3W for four doses	453 453	56 (19-83) 54 (18-86)	4
	Robert J. Motzer 2015 NCT01354431	Full text	Blinded, randomized, multicenter phase II trial	Renal Cell Carcinoma	Metastatic Renal Cell Carcinoma	1. Nivolumab 0.3mg/kg Q3W 2. Nivolumab 2mg/kg Q3W 3. Nivolumab 10 mg/kg Q3W	60 54 54	61±9 61±8 61±10	3
	Y.-J. Bang 2018 NCT02625623	Full text	multicentre, international, randomised, open-label, phase III trial	gastric cancer	metastatic gastric cancer/gastrooesophageal junction cancer	1. Avelumab 10mg/kg Q2W 2. Physician's choice of chemotherapy	185 186	59 (29-86) 61 (18-82)	3
